# Astrophysicists’ Conversational Connections on Twitter

**DOI:** 10.1371/journal.pone.0106086

**Published:** 2014-08-25

**Authors:** Kim Holmberg, Timothy D. Bowman, Stefanie Haustein, Isabella Peters

**Affiliations:** 1 School of Mathematics and Computing, University of Wolverhampton, Wolverhampton, United Kingdom; 2 Åbo Akademi University, Turku, Finland; 3 Dept. of Information and Library Science, Indiana University, Bloomington, Indiana, United States of America; 4 École de bibliothéconomie et des sciences de l’information, Université de Montréal, Montreal, Canada; 5 ZBW Leibniz Information Center for Economics and Christian Albrechts University Kiel, Kiel, Germany; Max Planck Society, Germany

## Abstract

Because Twitter and other social media are increasingly used for analyses based on altmetrics, this research sought to understand what contexts, affordance use, and social activities influence the tweeting behavior of astrophysicists. Thus, the presented study has been guided by three research questions that consider the influence of astrophysicists’ activities (i.e., publishing and tweeting frequency) and of their tweet construction and affordance use (i.e. use of hashtags, language, and emotions) on the conversational connections they have on Twitter. We found that astrophysicists communicate with a variety of user types (e.g. colleagues, science communicators, other researchers, and educators) and that in the ego networks of the astrophysicists clear groups consisting of users with different professional roles can be distinguished. Interestingly, the analysis of noun phrases and hashtags showed that when the astrophysicists address the different groups of very different professional composition they use very similar terminology, but that they do not talk to each other (i.e. mentioning other user names in tweets). The results also showed that in those areas of the ego networks that tweeted more the sentiment of the tweets tended to be closer to neutral, connecting frequent tweeting with information sharing activities rather than conversations or expressing opinions.

## Introduction

Astrophysics and astronomy are examples of academic disciplines that engage with the public and with scholars across disciplines to identify novel objects and recurring patterns in large data sets to help answer research questions. According to NASA [1], citizen scientists have helped answer “serious scientific questions” and provided the astronomical community with “vital data.” Christian, Lintott, Smith, Fortson, and Bamford [2] describe citizen scientists as being actively involved in achieving real research objectives. Projects related to astronomy and astrophysics like Galaxy Zoo (http://www.galaxyzoo.org), with more than 250,000 volunteers classifying galaxies (http://authors.galaxyzoo.org), and the Milky Way Project (http://www.milkywayproject.org), highlight the communications between researchers in astrophysics as well as astronomy and a broader audience that can include non-experts, volunteers, and collaborators from outside disciplines. As Kouper [3] notes, there is a “range of new ways of engaging the public in dialog and decision making… (that) have been introduced in practice and scholarly literature.” One way for scholars to engage both other scholars and groups participating in citizen science at large is through the use of social media applications.

An example of a social media application that has been shown to be of use to scholarly communication and citizen science is the blog. Bloggers use the medium to communicate their feelings, thoughts and reaction to matters of interest [4]. Scholars have been found to use blogs in order to “provide authoritative opinions about pressing issues in science… (and) because of their freewheeling nature, these blogs take scientific communication to a different level” [5]. In other scholarly communication discourse, research has shown that scientists who have blogs tend to discuss recent publications, socially relevant information, high-quality science, and that they write in a manner in which the information is useful to both academics and non-academics [6]. Niset [7] argued that “scientists… must strategically ‘frame’ their communications in a manner that connect with diverse audiences” and that scholars should no longer assume that simply bringing the public updated information about scientific facts is enough; instead, scholars should engage the public’s “values, interests, and worldviews.” The contributions of the contemporary scholar can be found in blogs, but also in other social media. Another context in which scholarly communication is occurring is in the microblogging site Twitter, where communication and interaction with a general audience are possible. The number of Twitter users grew by 39% from September 2012 to September 2013 (http://www.sec.gov/Archives/edgar/data/1418091/000119312513400028/d564001ds1a.htm), currently reaching 230 million active users, who post 500 million tweets per day (https://business.twitter.com/whos-twitter). It is the scholarly activity within this medium that is of interest to this work.

It has been suggested that traces of scholarly activities and conversations left in social media might convey something about the impact or visibility of scholarly research. Altmetrics is the research area that investigates these possibilities. While altmetrics still lacks a widely agreed definition, the concept is typically used to describe the measurement of impact or visibility of scientific articles and other scholarly activities in social media such as blogs, tweets, Facebook ‘likes’ and social bookmarks [8]. While scholars are using many different social media sites for scholarly communication, Twitter seems to be one of the most promising contexts in which to perform altmetric research because it contains more scientific content than many other social media sites [9]. Twitter is also the second largest source – behind the social reference manager Mendeley – of altmetric data that can be currently collected [9], [10]. Many papers [11], [12], [13] discuss Twitter’s usefulness for scholarly communication, particularly in terms of distributing information to a wider audience of researchers and the general public. Data can be collected via Twitter’s API and filtered in great detail by taking advantage of the API documentation and metadata that is included with the data. However, the content of the activities and the context in which these traces of research activities are created on Twitter are still relatively uninvestigated areas, even though they impact the validity of altmetrics. This raises several questions about altmetrics and the value it provides to the scientific community, including: Are the traces created in tweets conversations among scholars or do they represent activities where science is communicated to a wider audience? Does the content of these interactions reflect research activities of the tweeters? Does tweeting activity or publishing activity of the researchers have an impact on how and for what purpose they use Twitter? With whom do researchers interact with on Twitter?

To increase our collective understanding of the context in which potential altmetrics indicators are created on Twitter we need more research about how social media is used and perceived by researchers. In order to better understand the impact and context of scholarly communication on Twitter, this paper maps the conversational connections of a group of astrophysicists using Twitter and analyzes the content of their tweets. Active engagement with the public (i.e. citizen scientists) and active use of social media (see http://www.wired.com/2013/11/nasa-socials) makes astrophysicists an interesting group to study Twitter use for scholarly communication. Because of this, the results may not be generalizable to other disciplines, but rather present a particular case. In this paper we seek to answer the following three research questions:

1. With whom do astrophysicists have conversational connections on Twitter? Who do they mention in their tweets?
2. Does traditional scholarly communication (i.e., publication frequency) affect conversational behavior of astrophysicists (i.e., tweeting frequency, with whom they talk)?
3. Do tweets of astrophysicists show syntactical and linguistic particularities, like intensive use of hashtags or emotions?

Our hypothesis is that the contexts in which attention and visibility are created in Twitter and the intended audience of that content have impact on whether altmetrics can be used to evaluate influence or visibility of scholarly communication. By mapping who the astrophysicists mention in their tweets we will learn about the intended audience of astrophysicists Twitter communication and by answering research questions 2 and 3 we will learn more about the context in which the tweets have been published.

## Literature Review

It has been reported that social media, like blogs and Twitter, are used in academia “at all points of the research lifecycle, from identifying research opportunities to disseminating findings” [14]. Scholars have reported both being familiar with a diverse set of social media tools and that they would like to increase their usage of these tools in the future [15]. However, different surveys have reported very different usage figures. Rowlands et al. [14] reported that around 80% of researchers used social media in research, while 66% of the 939 professors in the study by Moran, Seaman and Tinti-Kane [16] reported using social media in the past month. In these studies social media was defined rather broadly to include tools for conferencing and collaborative authoring, which may explain the high percentage of usage.

### Twitter uptake and use

Recently Twitter has become one of the most popular social media sites [17], but while the coverage of scientific content is higher on Twitter than on many other social media sites [9], the actual use of Twitter in scholarly communication still remains low. According to Rowlands et al. [14] and Priem, Costello and Dzuba [18] respectively, 9.2% and 2.5% of scientists are active on Twitter. Haustein et al. [19] found that among 71 surveyed participants at a bibliometric conference in 2012, 43.7% had a Twitter account. These were mostly used for private reasons, to connect with people professionally, to distribute professional information and to improve one’s visibility on the web. Bowman et al. [20], who surveyed over 200 Digital Humanities scholars, found that 80% of respondents rated Twitter as a relevant source of information for digital humanities research and 73% rated Twitter as relevant for dissemination of research information. In a recent survey Pscheida et al. [21] asked a representative sample of scholars at German universities about social media use and found that 15.2% of 778 (cases were weighted according to type of university and region to account for particular over- and underrepresentation in the sample.) participants used Twitter. Of those 118 (weighted) who reported using Twitter, 30.5% reported using it only for private communications while 69.5% used it occasionally in a professional context. In addition, 22.0% of respondents reported using Twitter daily and 73.2% used it at least once per week. Because Twitter was known by 97.2% of the German survey respondents, Pscheida et al. [21] conclude that the microblogging platform is rather a hype medium that is mostly spoken about but rarely used in scholarly communication (at least in Germany). The variation from 2.5% to 80% in Twitter uptake among scholars is influenced by both the differences between scientific disciplines [13] and the time when the survey was conducted. However, both surveys including all fields of science suggest that work-related Twitter use among scholars remains low at around 10%.

### Twitter affordances

Although Twitter is not designed as a social network where users are connected via mutual relationships, user networks and conversations do evolve through Twitter-specific affordances like following another Twitter user, retweeting someone’s tweets, mentioning usernames in the tweets and by using the same hashtags. Users can subscribe to another user’s Twitter time line by following his or her account (i.e. forming a directed relationship between the follower and the followee). Another distinctive affordance available to users is the retweet. A retweet occurs when a user redistributes another’s tweet. Because of the specific format of this affordance, one can detect so-called pure retweets (e.g. tweets that start with ‘RT’) in order to analyze how information is disseminated and forwarded through the Twitter networks. In addition to the retweet, Twitter also affords users the ability to direct tweets at other users through the use of an ‘@’ symbol followed by a username. Honeycutt and Herring [22] have shown that the use of the *mention* affordance is a strong indication that the tweet is conversational in nature as about 90% of the tweets containing *@username* were found to address a user as part of a conversation. Honeycutt and Herring [22] further discovered that about one third of all tweets in their study contained a username, thus being conversational. In a way, Twitter has become the digital water cooler around which users discuss their work [23]. In addition to retweets and mentions, users also make use of the hashtag affordance to categorize, organize, and retrieve tweets. The use of hashtags is extremely popular during major events (e.g., televised events such as the #royalwedding), natural disasters (e.g., #tsunami [24]), or scientific conferences (e.g., #asist13 [25]). As such, hashtags may resemble the traditional function of metadata by enhancing the description and retrievability of documents.

### Sentiment of Tweets

The linguistic construction of tweets, especially the use of emotional-laden terms, may also affect conversations and a tweet’s dissemination. Tweets containing strong sentiments are found to be retweeted more often than neutral tweets [26], [27], which leads to the assumption that emotional tweets are more likely to be widely distributed. The level of activity or experience, in terms of the number of tweets posted by a Twitter user, has not been found to influence the sentiment of the tweets (i.e. sentiment of tweets from more active users do not differ from the sentiment of tweets from anyone else [26]). It has, however, been shown that adding positive emoticons to tweets is very common and that, at least in one case, 85% of a particular set of tweets had positive sentiments [28], [29]. However, Thelwall [30] came to a contradictory conclusion by discovering that sentiments are barely expressed in tweets finding that the sentiment of tweets did not change even when the covered event turned out to be very negative. Hence, Thelwall [30] concluded that sentiment analysis is not able to properly detect linguistic phenomena like sarcasm and irony from messages of limited lengths like tweets.

Given that we consider tweets as medium for scholarly communication we have to look at work discussing the expression of sentiment in scientific publications. Verlic, Stiglic, Kocbek and Kokol [31] analyzed frequently used strong adjectives and adverbs in a five-year span of conference papers to detect enthusiastically and passionately presented research results. They concluded that: “we could not claim that sentiment as defined in scope of our study is obviously present in the papers we analyzed.” Small [32] published an exploratory study on how attitudes towards cited work were expressed in co-citation networks finding that sentiments were not understood as positive or negative emotions but as structural terms for argumentation (e.g., discovered, demonstrated) or description of scientific results (e.g., approaches, fundamental). He showed that sentiments vary in citation contexts of different disciplines and “provide insights into the current issues and concerns of a research community” (p. 387). There have been a few others [33], [34] who have looked at this phenomenon, but overall the research in that area is sparse. Because the research is sparse, Twitter continues to be a promising context to study given the findings of former analyses of sentiments and distribution patterns on Twitter [35].

### Twitter as an altmetric source

Earlier studies examining the impact or visibility of research using traces of scientific activities in social media have discovered a correlation between altmetric indicators and more traditional measures of scientific impact such as citation counts [36], [37], although more recent findings have questioned this correlation. A large-scale study [10], [38] based on 1.4 million PubMed papers found that correlations are generally very low and vary by scholarly sub-discipline as reflected in Spearman values of citations and tweets ranging between −0.200 (Speech-Language Pathology & Audiology) and .327 (General & Internal Medicine). It has also been shown that results may be significantly impacted by the time of tweeting and time of article publication as “comparisons between citations and metric values for articles published at different times, even within the same year, can remove or reverse this association” [9]. Another issue worth mentioning here concerns the different versions of the same publication or the same research that may exist (e.g., preprint, conference proceeding and journal article) and that all can receive altmetrics. Should all of these altmetrics be aggregated or should they be treated separately? Haustein and colleagues [39] combine tweets mentioning the arXiv e-print and the paper published in the journal of record handling the as two versions of the same document, but it could very well be argued that various versions represent different publications, particularly if significant changes were made during the review process.

Haustein, et al. [40] discovered that, for a set of astrophysicists on Twitter, those that were more active on Twitter (i.e. published more tweets) published less scientific articles and vice versa; this negative correlation between tweeting activity and publishing activity may have significant impact on the reliability and generalizability of altmetric measures. Those researchers that do not actively participate in social media to promote and discuss their own work may be left in a disadvantaged position compared to those researchers that actively engage in online communication. This also raises the question of gaming the altmetrics; when is one only promoting his or her own work and when is it considered as gaming the numbers to intentionally inflate one’s online visibility? It is also possible that if the altmetrics are created by a certain type of users (those that are active in social media) it may undermine the generalizability of the altmetrics. It is clear that more research is needed to investigate the content and context in which scholars use Twitter and what role it plays in scholarly communication. This work addresses this need by examining tweets and Twitter conversations of a sample of astrophysicists.

## Methods and Data Presentation

**Figure 1:**
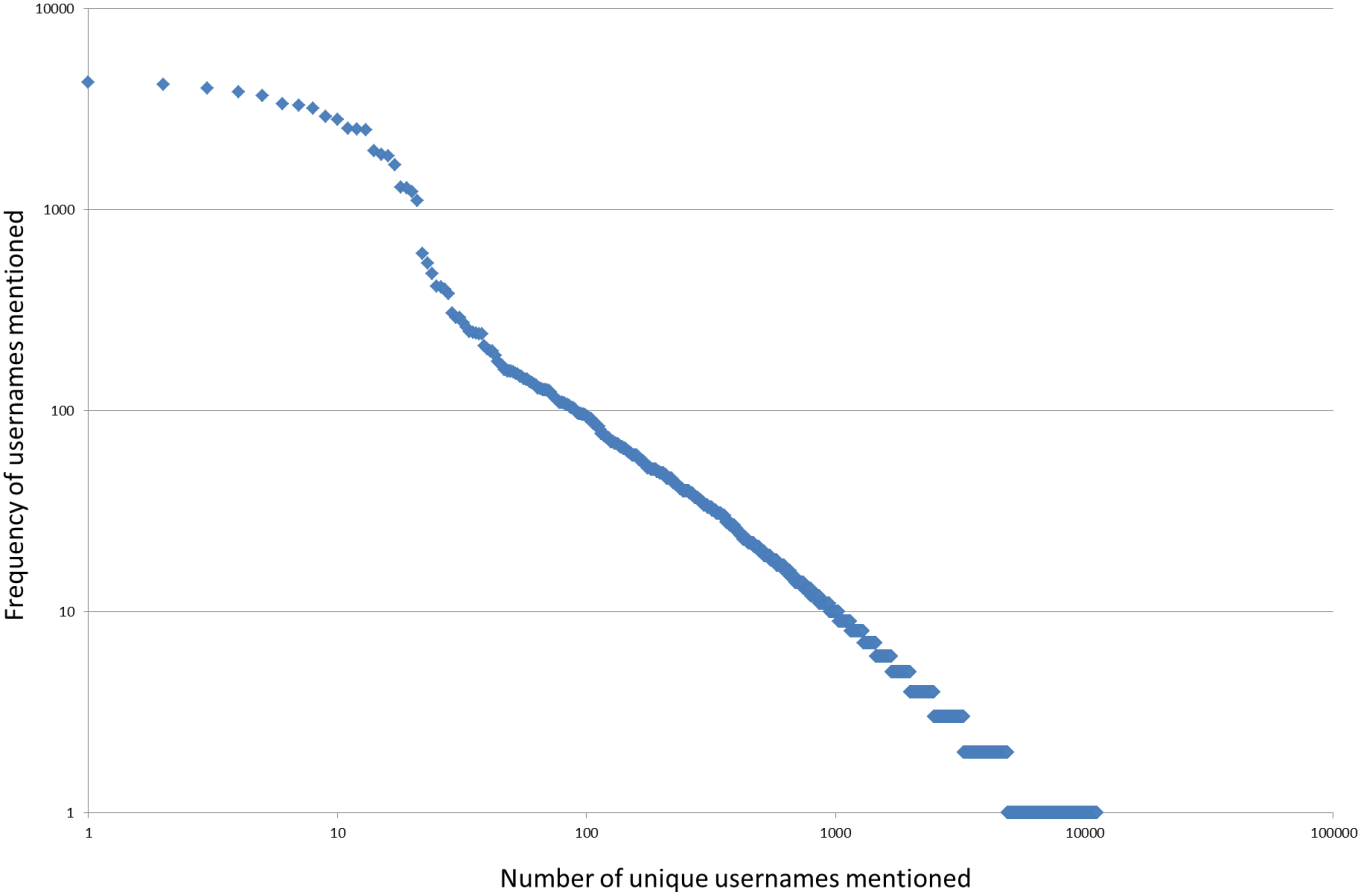
The frequencies with which usernames were mentioned on a log-scale. Figure 1 shows how skewed the frequency with which usernames were mentioned was, with a few usernames that were mentioned frequently and with a lot of usernames that were only mentioned once or just a few times.

A total of 68,232 tweets published by 37 astrophysicists were retrieved through the Twitter API in May 2013. The 37 astrophysicists represent a wide selection of astrophysicists, with great variation in both publishing and tweeting activity. They also represent different levels of academic seniority. A more detailed description of the sample of astrophysicists and tweets can be found in Holmberg and Thelwall [13] and Haustein et al. [40]. The 37 astrophysicists mentioned a total of 11,252 unique usernames in their tweets and each username was mentioned on average 10 times, while the median for the whole dataset was 1; this indicates highly skewed data (Figure 1).

In order to investigate who the astrophysicists approach or mention in their tweets we created an ego network map of the tweet authors and the usernames they mentioned. As we neither know whether these tweets were part of a dialogue or conversations between the astrophysicists and the other users, nor is it the purpose of this paper to investigate the full communication network of the astrophysicists, we will call these connections between the astrophysicists and the usernames they mention ‘conversational connections’. A conversational connection therefore indicates that a conversation has been initiated or that a certain username has been approached or at least mentioned by one of the 37 astrophysicists investigated in this research. The 37 astrophysicists mentioned a total of 11,252 usernames in their tweets, creating 56,415 conversational connections between the astrophysicists and the other users mentioned in their tweets. If tweets by the astrophysicists mentioned more than one username, then all usernames were extracted and treated as co-mentions of source-target pairs (i.e., astrophysicist1– username_1; astrophysicist1– username_2, …, astrophysicists1– username_n). Webometric Analyst software [41] was used to create a conversational network based on these conversational connections and Gephi software [42] was used for network visualization.

The network was limited to users that were mentioned or that had tweeted 20 or more times. For the final analysis we included 32 astrophysicists (tweet authors) as well as 511 usernames that were mentioned in the tweets. Because the 32 astrophysicists were in contact with 511 people and the groups overlapped, 518 of the most mentioned usernames are represented in the conversational network. The underlying matrix thus contained 32 rows and 518 columns, where the cells contained the number of conversational connections with a minimum of 20 occurrences. By using a small set of astrophysicists as a seed set, we also made sure it was possible to manually code users in order to find out with whom these astrophysicists actually communicate on Twitter. The usernames in the conversational network were coded according to their role or professional titles as *32 astrophysicists* (the seed dataset), *amateur astronomers*, *corporative*, *organization or association*, *other astrophysicists*, *other researchers*, *science communicators*, *students*, *teachers* or *educators*, *other* or *unknown* based on information found directly in their Twitter profile or by following provided links. For instance, the *science communicators* category included science bloggers and science journalists, while the *organizations and associations* category included organizations related to astrophysics or astronomy (e.g. NASA, ESA and ESO). *Others* included users that could not be coded into the other categories and *unknown* included those users whose role or profession could not be determined due to lack of information in their Twitter profiles or links. The categories were created inductively, thus new categories were created when users did not fit into existing categories. The coding was carried out by one of the authors. Gephi’s community detection [43], [44] was used to detect more densely clustered groups of users based on the number of connections between them. The content of the conversations and the professional makeup of these clusters were analyzed in order to learn more about the conversational connections of the astrophysicists.

In addition to the conversational connections, we also analyzed the content of the tweets and the use of hashtags to determine both popular hashtags used by astrophysicists and whether hashtag sharing among astrophysicists leads to the development of online communities. We defined a hashtag as any string of characters between a ‘#’ symbol and a blank space (e.g., #NASA) and automatically extracted all such occurrences from the tweets. Poorly constructed hashtags (e.g., # (blank space) term) were not captured for this analysis. The hashtags were analyzed according to the communities detected in the graph of ego networks. In order to learn more about the content of conversations in the conversational clusters that were detected with Gephi, we used VOSviewer [45] to extract noun-phrases from the tweets. VOSviewer applies a linguistic filter based on a part-of-speech tagger, which extracts noun phrases and merges regular singular and plural forms [45]. We included all the noun-phrases in the analysis, thus no thresholds or relevance scores offered by VOSviewer were applied to restrict the results. The similarities between the noun phrases used in each cluster were measured using Pearson’s *r*.

The SentiStrength tool [35] was used to determine the sentiments of tweets in each cluster. SentiStrength uses a lexical approach with sentiment-word-lists as well as several rules to process linguistic variation in terms. The tool was especially constructed for analysis of short texts found on the (social) Web (e.g., taking into account exorbitant use of punctuation [35]). The sentiment analysis results provide two scores for each analyzed word (i.e. negative and positive) that ranged between −5 and −1 and 1 and 5 respectively. Score of 1 and −1 indicate that the word is neutral and has no sentiment. The mean positive and mean negative values as well as a combined Sentiment Score (i.e. negative values plus positive values) for the tweets in each cluster were measured and compared.

## Results

**Figure 2:**
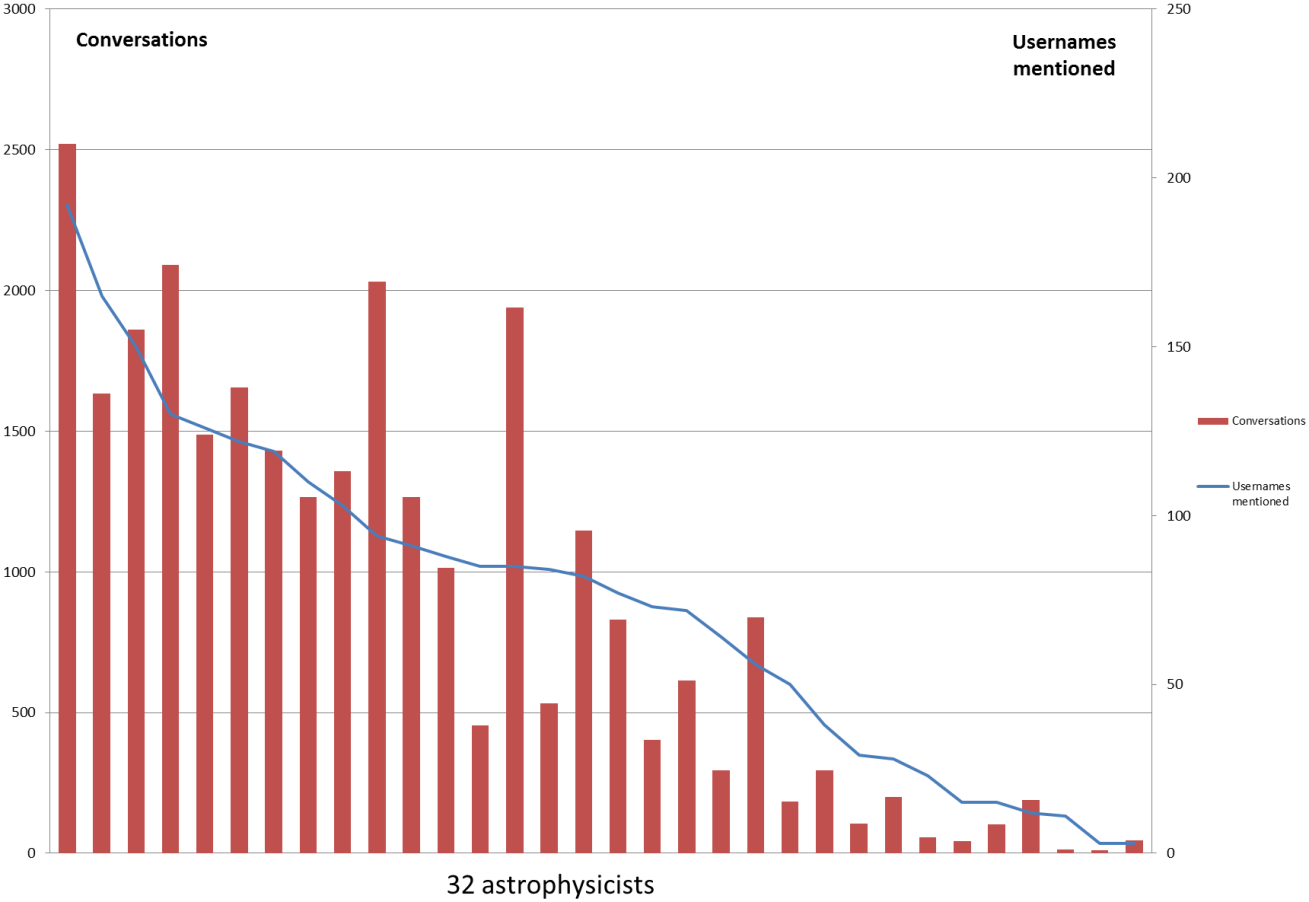
Number of people contacted and the number of conversations had by the 32 astrophysicists. Figure 2 shows the number of conversations the studied astrophysicists had with other usernames and the number of unique usernames they mentioned. Overall there is a strong correlation between the number of users mentioned and the number of conversations, some astrophysicists have more conversations with fewer Twitter users while others have their conversations with a much wider audience.

The 518 nodes (representing the 32 astrophysicists and the usernames they mentioned in their tweets) were connected with each other through 2,395 edges resulting in a set of 27,923 conversations (it should be noted that if astrophysicist1 mentioned two other people in a single tweet, that counted for two conversational connections). Figure 2 shows the number of people the 32 astrophysicists mentioned in their tweets and the number of conversational connections they had with these users. The frequency with which the astrophysicists mentioned other usernames varied a great deal; results fell along a continuum between over 2,500 conversational connections with almost 200 different usernames, to only a few conversations with a few users. Overall there is a strong correlation between the number of users mentioned and the number of conversations (0.94 with Spearman rank correlation), but this correlation and Figure 2 also indicate that some astrophysicists have more conversations with fewer users while others have their conversations with a much wider audience.

For clarity the 32 (6.18%) astrophysicists whose tweets we retrieved were simply labeled as “32 astrophysicists” (Table 1), the other usernames were coded according to their role or professional titles. Most of the users mentioned in the tweets by astrophysicists were coded as science communicators (24.13%), other astrophysicists (21.62%), organizations or associations (13.32%) and others (11.20%). A perhaps surprisingly low number of the users mentioned were teachers, students, or amateur astronomers. Especially with the large number of citizen scientists involved in various projects related to astrophysics we would have expected astrophysicists to interact with amateur astronomers on Twitter and thus see more mentions of non-scientists.

**Table 1:** Roles of the users mentioned in the tweets.

Role or profession	
Science communicator	24.13%
Other astrophysicists	21.62%
Organization or association	13.32%
Other	11.20%
Unknown	8.11%
32 astrophysicists	6.18%
Other researchers	5.98%
Teacher or educator	3.67%
Corporative	2.32%
Students	2.12%
Amateur astronomer	1.35%

**Figure 3:**
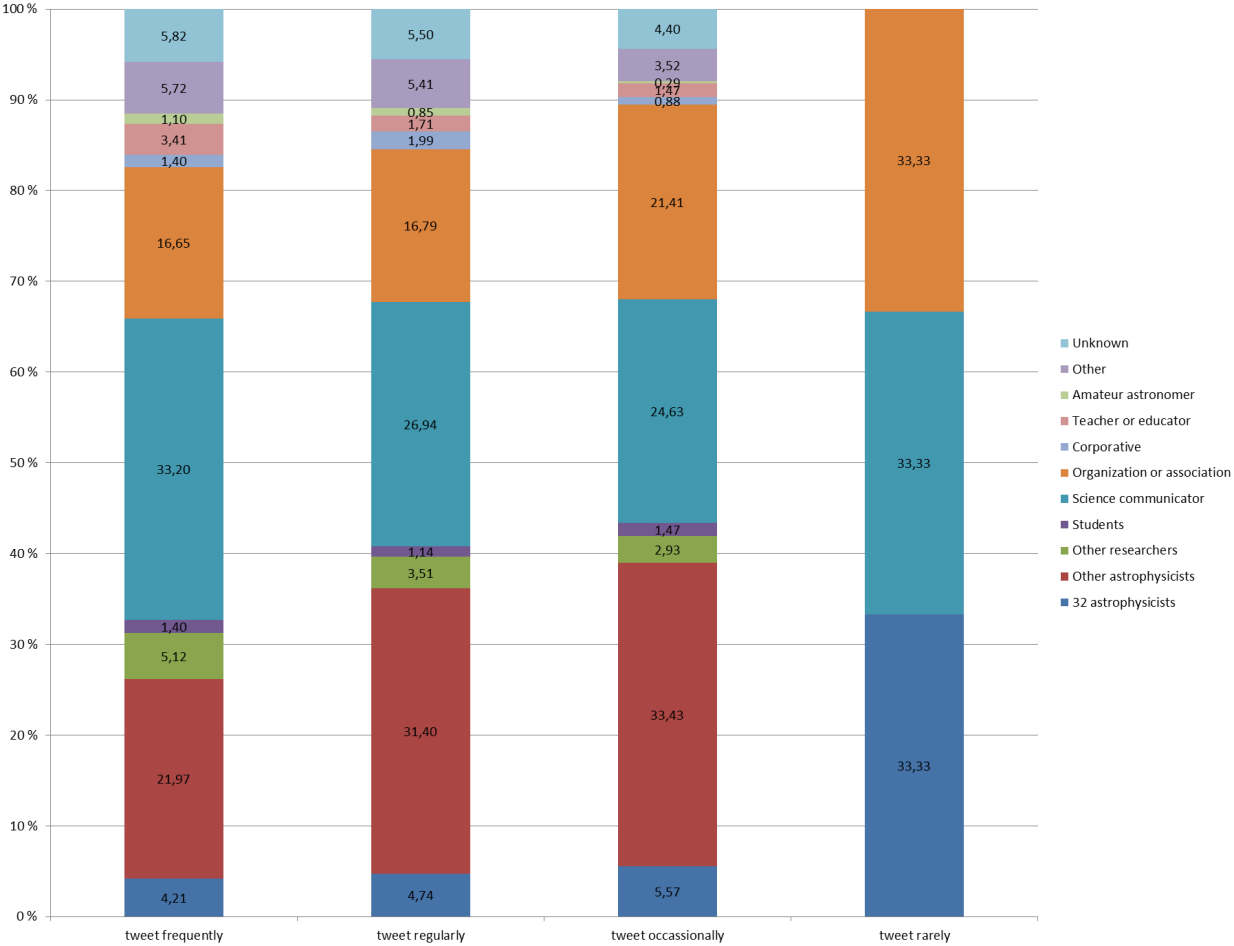
Percentage of people mentioned by role by astrophysicist on average by tweeting behavior. Figure 3 shows the conversational connections to users with different roles or professions according to the tweeting activity of the astrophysicists.

In another work [40], the astrophysicists were categorized according to their tweeting activity and their publishing activity. We used these categories to analyze whether tweeting activity or publishing activity had an impact on conversational connections in Twitter. Of the 32 astrophysicists qualified for this analysis, 10 tweeted frequently, 11 tweeted regularly, 10 tweeted occasionally and only one tweeted rarely. Because we limited this analysis to those usernames and those astrophysicists that were mentioned at least 20 times, the number of astrophysicists in the last category remained low. Based on the categories in Haustein, et al. [40], 6 astrophysicists do not publish, 9 publish occasionally, 13 publish regularly, and 4 publish frequently. In Figure 3 the conversational connections to users with different roles or professions are grouped according to the tweeting activity of the astrophysicists. The results indicate that one third of the usernames mentioned by the 10 astrophysicists who tweet frequently were science communicators, while about one fifth were other astrophysicists. The profiles of the conversational connections for the astrophysicists who tweet regularly and who tweet occasionally are fairly similar, with about one third of the mentioned usernames being other astrophysicists and about a quarter being science communicators. Only one astrophysicist was classified to the group that tweeted rarely; because of this lack of data the conversational profile for this group cannot be considered as representative. Only 3 different usernames were mentioned in 11 tweets by the single astrophysicist in this group.

**Figure 4:**
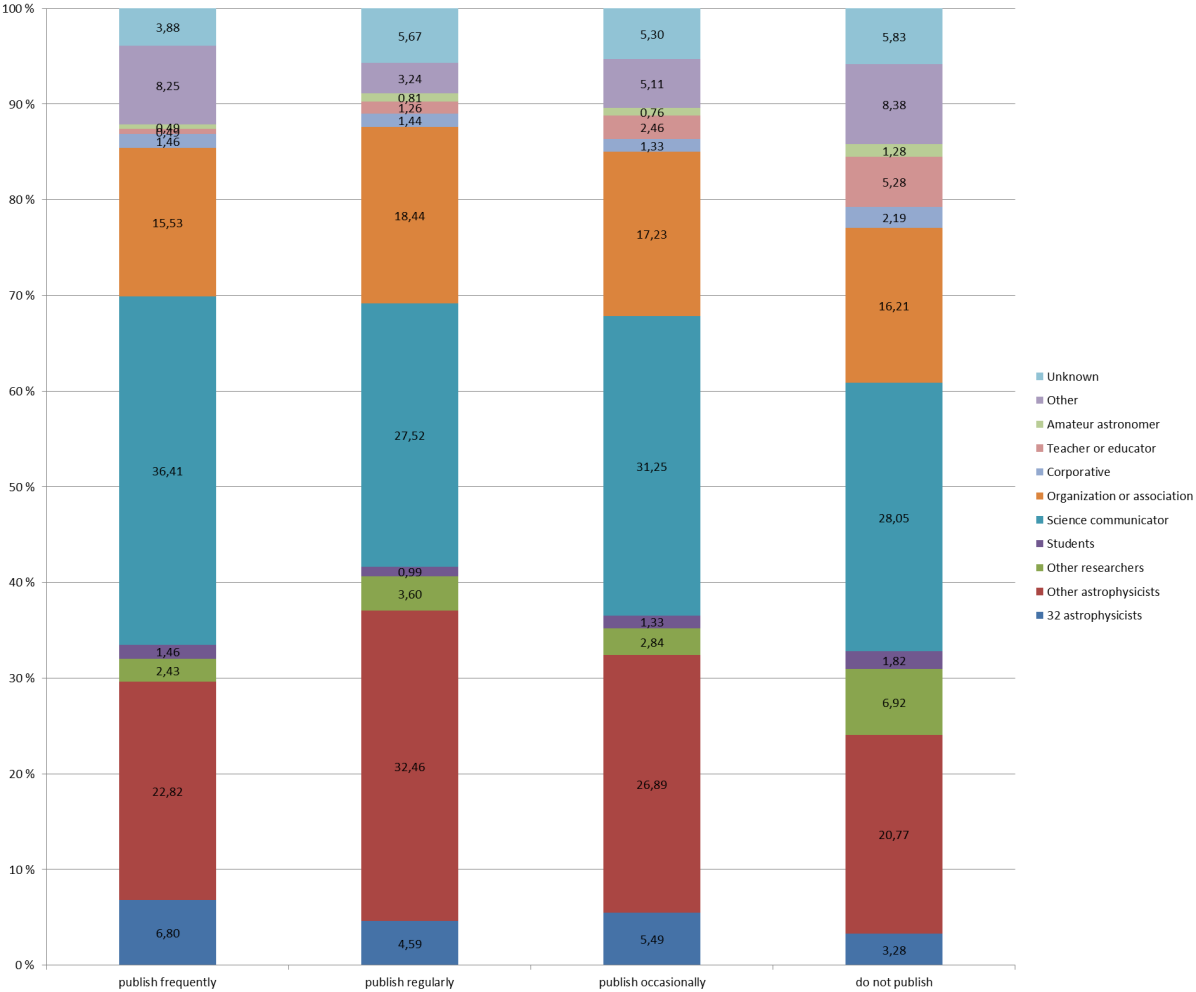
Average of people mentioned by role by astrophysicist by publishing behavior. Figure 4 shows the conversational connections to users with different roles or professions based on the publishing behavior of the studied astrophysicists.

The conversational connections based on publishing behavior were also investigated (Figure 4). Those that publish frequently had the most conversational connections to science communicators (36.4%), while the science communicators mentioned in the other groups were between about 27.5% and 31.3% of the total amount of conversational connections. Another group of frequently mentioned Twitter users were the other astrophysicists; these were mentioned most by those that publish regularly (32.5%), while in the other groups they counted for between 22.8% and 26.9% of the mentions. Various organizations and associations related to astrophysics or astronomy (e.g. NASA, ESA) counted for between 15.5% and 18.4% of the mentions in all categories. All the remaining roles received some references, but clearly less than the above-mentioned roles.

**Figure 5:**
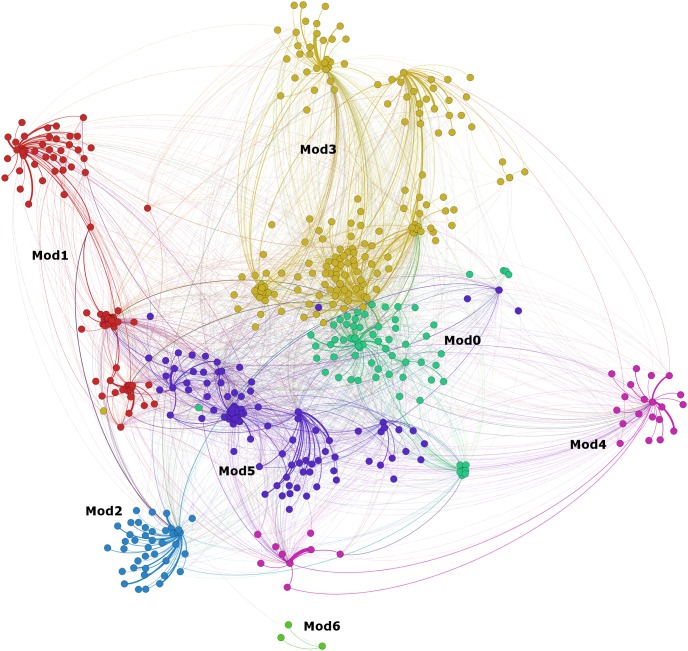
Conversational connections in the astrophysicists’ tweets. The network graph in figure 5 shows the conversational connections of the astrophysicists and the communities in them as detected with Gephi’s community detection.

We were also interested to map who the astrophysicists mentioned in their tweets. To map the whole communication network around the astrophysicists was beyond the scope of this research, as we wanted to investigate with who the astrophysicists initiated conversations with and who their intended audience were. The conversational connections between the 32 astrophysicists and those they mentioned in their tweets were visualized in a network map (Figure 5) using the OpenOrd layout [46]. The map shows the ego networks of the 32 astrophysicists, created from the outgoing connections (usernames mentioned) in their tweets. The overall graph-clustering coefficient of the network was 7.870, density was 0.018, and average distance was 2.604. Although the density of the graph was fairly low, the graph had high clustering and short distances between the nodes. Both features are frequently connected with the small world phenomenon [47]. A community detection [43], [44] in Gephi revealed seven clusters of frequent interactions in the graph. These were colored and indicated as Mod0–Mod6 in Figure 5. The clusters vary in size, as they range from the smallest cluster with only 3 users (Mod6) to the largest with 180 users (Mod3). There was also some overlap and interaction between the different clusters.

**Figure 6:**
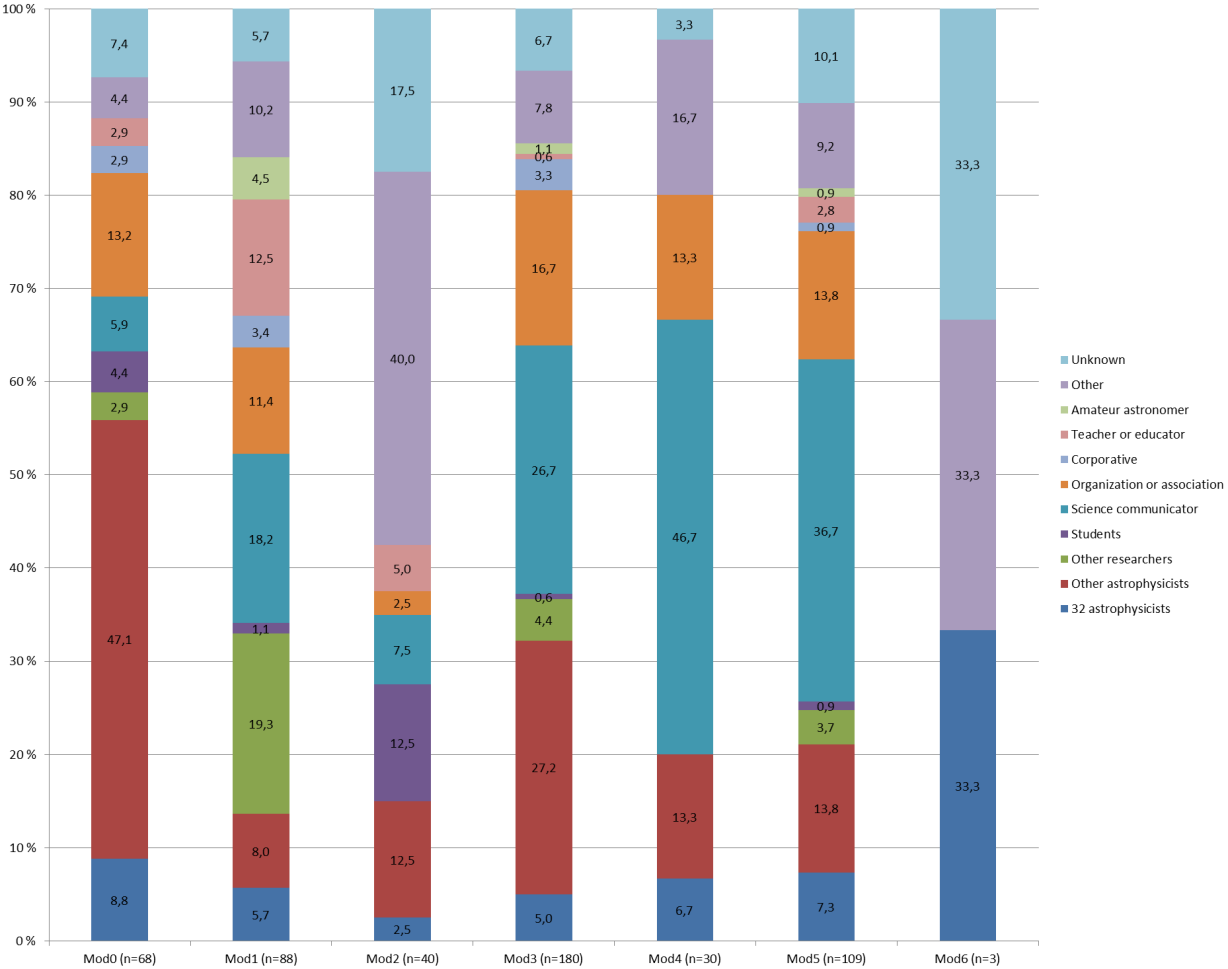
Percentage of people with different roles in the 7 communities. Figure 6 shows the professional make-up of the communities detected in the conversational network. The results show how the different conversational communities consist of very different types of users.

The conversational connections within these clusters were analyzed and differences between them were discovered (Figure 6). The astrophysicists in the first cluster (Mod0) mostly mentioned other astrophysicists (47.1%), while the astrophysicists in Mod4 mostly mentioned science communicators (46.7%). Astrophysicists in Mod2 mentioned students (12.5%) and teachers (5.0%) more than other clusters, suggesting that these astrophysicists use Twitter more for educational purposes. However, this group of astrophysicists also had the most connections to other Twitter users (40.0%) and to unknown users (17.5%). The astrophysicists in Mod1 had the most connections to other researchers (19.3%), possibly indicating a multidisciplinary component in their research. All clusters, except Mod6, had conversational connections to almost all categories of Twitter users, again demonstrating the variety of connections that the astrophysicists have on Twitter. It should be noted, however, that Mod6 only contains three Twitter users, one of the observed astrophysicists and two usernames he or she mentioned in tweets.

The content of the conversations within these clusters was analyzed by extracting the noun phrases from the tweets. The noun phrases from the last cluster, Mod6, were excluded from the analysis due to the significantly lower number of tweeters. Among the most frequently used noun phrases or words in the clusters were words related to time (e.g. *time*, *day*, *today*, *week*, *year*) and general astrophysics terms (*star*, *planet*, *earth*, *moon*, *mars*, *science*). The similarities in the use of noun phrases and words between the clusters were measured with Pearson’s *r* (Table 2). The results indicate modest to high similarities in the choice of words and noun phrases in the clusters, as similarities range between 0.41 (Mod2 and Mod5) to 0.78 (Mod0 and Mod3). Interestingly, the composition of the mentioned usernames is very different for clusters with high similarities and with low similarities. The highest similarity was between clusters Mod0 and Mod3 even though the roles of mentioned usernames as identified through their profile information are very different (Figure 6).

**Table 2:** Similarities (Pearson's *r*) between the noun phrases used in each community (Mod0–Mod6).

	Mod0	Mod1	Mod2	Mod3	Mod4	Mod5
**Mod0**		0.66	0.55	0.78	0.74	0.60
**Mod1**	0.66		0.68	0.73	0.70	0.64
**Mod2**	0.55	0.68		0.55	0.59	0.41
**Mod3**	0.78	0.73	0.55		0.72	0.75
**Mod4**	0.74	0.70	0.59	0.72		0.68
**Mod5**	0.60	0.64	0.41	0.75	0.68	

The hashtags found in the tweets in the different clusters (Mod0–Mod6) were also analyzed separately from the tweet content. Researchers in the different clusters used hashtags very differently; hashtag use ranged from a total of 7 unique hashtags used 11 times in Mod2 to over 1,000 unique hashtags used almost 4,000 times in Mod1, Mod 3 and Mod5 (Table 3). The clusters labeled as Mod1, Mod3 and Mod5 were also very similar in their choice of noun phrases (Table 2), but the username roles mentioned in Mod1 were clearly different from Mod3 and Mod5 (Figure 6).

**Table 3:** Number of hashtags and unique hashtags used in the tweets of the detected communities.

Mods	Total hashtags	Unique hashtags
**Mod0**	2,569	633
**Mod1**	3,748	1,215
**Mod2**	321	184
**Mod3**	3,977	1,074
**Mod4**	1,656	564
**Mod5**	3,862	1,350
**Mod6**	11	7

To gain a deeper understanding of the content of the tweets sent in each cluster, the background and meaning of the five most used hashtags from each cluster were investigated (Table 4). The most frequently used hashtags in the clusters labelled Mod0, Mod1, Mod2 and Mod4 contained hashtags related to astrophysics or astronomy; some of the hashtags are used only by a single tweeter to label their tweets and to distinguish the tweets related to astrophysics from their other tweets (#twinkletweet, #AstroFact), while some are related to functionality and use of Twitter in general (#FF, #fb). The most frequent hashtag in Mod 0 reflects parallel use of Twitter and Facebook, presumably within the “Astronomers” group (https://www.facebook.com/groups/123898011017097), which only allows professional astronomers to join. This professional focus is corroborated by the fact that 47% of all mentioned Twitter users of Mod0 were coded as other astrophysicists and it also contains the highest share of the 32 astrophysicists (except for Mod6). Mod1 and Mod2 reveal more personal interactions and conversations with students. For example, the most frequent cluster-specific noun phrases (i.e., terms appearing in only this and maximum one other cluster) in Mod2 are “hug”, “hahahaha”, “cake”, “revision” and “tea”, while Mod1 contains specific terms of a professor who blogs about astronomy, physics and pop culture, often featuring his children. Mod1 also mentions teachers, amateur astronomers and other researchers more frequently than other clusters. Discussions in Mod3 are related to science policy, and more specifically science programs and funding cuts in the UK and the Science & Technology Facilities Council (STFC) as indicated by the top five hashtags #stfc, #scipolicy, #rcuk, #scienceisvital and #scicuts as well as the most frequent cluster-specific noun phrases “programme”, “stfc”, “item” and “deadline”. The UK focus also demonstrates a geographic connection between the tweeters in Mod3. Hashtags in Mod5 are related to the Hubble (#Hubble) and James Webb Space (#JWST) telescopes, NASA (#NASA) and mathematics (#math, #mathed). In addition, some of the hashtags from specific clusters are connected to specific conference or workshop (e.g., #aas221, #cs17, #astro101, #clickers2012, #scio13, #gzconf). It is possible that tweeting about conferences have had some impact on the formation of some of the clusters that were detected, emphasizing the fact that the clusters detected in this research consist of people that share similar interests.

**Table 4:** Top 5 hashtags and their meaning by community.

Cluster	Hashtag (frequency)	Explanation
Mod0	#fb (519)	Indicates tweets that are automatically imported to Facebook
Mod0	#twinkletweet (284)	A tag used by an astrophysics professor to distinguish his personal tweets from professional tweets
Mod0	#dotastro (139)	“Astronomy aims to bring together an international community of astronomy researchers, developers, educators and communicators to showcase and build upon these many web-based projects, from outreach and education to research tools and data analysis” (http://dotastronomy.com/about/)
Mod0	#aas221 (81)	American Astronomical Society 221st Program
Mod0	#cs17 (81)	17th Cambridge Workshop on Cool Stars, Stellar Systems and the Sun
Mod1	#AstroFact (348)	A tag used by an astronomy professor to tweet astronomy facts and distinguish these facts from other tweets
Mod1	#astro101 (142)	Colloquium at CAPER Center for Astronomy & Physics Education Research
Mod1	#clickers (105)	Clickers and other classroom technologies can enable institutions and faculty to respond to the transformation of the learning environment into an interactive space
Mod1	#clickers2012 (103)	Clickers Conference, 2012, Chicago
Mod1	#scio13 (86)	ScienceOnline2013, 7th annual international meeting on Science and the Web
Mod2	#gzconf (22)	Galaxy Zoo conference
Mod2	#FGM (19)	*Female Genital Mutilation*, Reaction *to* a campaign against FGM which was the subject of a Channel 4 documentary, The Cruel Cut, which features the shocking scenes where Leyla Hussein (co-founder of the anti-FGM charity Daughters of Eve) asks people to sign the petition.
Mod2	#hugs (18)	Expressing emotion
Mod2	#NHS (14)	National Health Service, UK
Mod2	#FF (13)	Follow Friday: Tweet the names of Twitter users you'd like others to follow and tag it with followfriday and/or FF
Mod3	#stfc (409)	Science & Technologies Facility Council, UK
Mod3	#scipolicy (216)	Science Policy, UK
Mod3	#rcuk (143)	Research Council UK
Mod3	#scienceisvital (122)	“We are a group of concerned scientists, engineers and supporters of science who are campaigning to prevent destructive levels of cuts to science funding in the UK” (scienceisvital.org.uk).
Mod3	#scicuts (94)	Belongs to #scienceisvital
Mod4	#AAS218 (188)	American Astronomical Society 218^th^ Program
Mod4	#PS1 (95)	PS1 Prototype Telescope on Haleakala, Maui.
Mod4	#NucATown (79)	Nuclear Astrophysics Town Meeting
Mod4	#FF (48)	Follow Friday: Tweet the names of Twitter users you'd like others to follow and tag it with followfriday and/or FF
Mod4	#astrojc (44)	Astronomy Twitter Journal Club where people meet up on Twitter at a prearranged day and time and discuss an interesting piece of astronomy research
Mod5	#Math (330)	Mathematics
Mod5	#JWST (256)	James Webb Space Telescope
Mod5	#nasa (202)	National Aeronautics and Space Administration
Mod5	#Hubble (154)	Hubble space telescope
Mod5	#mathed (152)	Mathematics education

A positive and a negative sentiment value and a combined sentiment score of the tweets from each cluster were measured (as shown in Table 5). Because it was discovered that 449 tweets did not have any content (or only contained hashtags), the total number of tweets used in the sentiment analysis was 67,783. None of the clusters showed strong positive or strong negative sentiments, as the mean values range between 1.825 and −1.509 (range of possible values being between 5 and 1 for positive values and −1 and −5 for negative values). The combined mean sentiment scores range between 0.316 (Mod2) and −0.071 (Mod4). The sentiment of the tweets in the clusters labeled as Mod0 and Mod2 were somewhat more positive, while the sentiment of the tweets in Mod4 and Mod5 were virtually neutral.

**Table 5:** Sentiment of tweets by communities.

Mod	Mean positive	Mean negative	Sentiment score	Sum of tweets
**Mod0**	1.596	−1.294	0.302	7481
**Mod1**	1.656	−1.468	0.188	12127
**Mod2**	1.825	−1.509	0.316	3255
**Mod3**	1.582	−1.337	0.245	17709
**Mod4**	1.386	−1.458	−0.071	6033
**Mod5**	1.448	−1.343	0.104	20735
**Mean**	1.582	−1.387	0.194	9683

Interestingly there was a negative correlation between the sentiment score of the clusters and the number of tweets sent. A Spearman correlation of −0.371 (p = 0.497) suggests that in the clusters where more tweets were sent the sentiment of the tweets were closer to neutral, while clusters where fewer tweets were sent the tweets were somewhat more positive.

## Discussion

The results of this work indicate that the astrophysicists in this study are in conversational connections with a wide variety of other Twitter users, although some difference in the usage can be identified. As noted earlier, Haustein, et al. [40] found that astrophysicists who tweet frequently do not publish frequently and vice versa. Our results indicate that astrophysicists who tweet frequently mention science communicators more than other astrophysicists and other researchers, which is a behavior differing from those who tweet less frequently. This suggests that astrophysicists who frequently tweet do so for reasons other than to communicate directly with colleagues. Interestingly, both frequent tweeters and frequent publishers often mention science communicators in their tweets. Although not confirmed by the results in this study it could be that those who publish frequently maintain more conversational connections to science communicators in order to disseminate research results to a wider audience, while those who tweet frequently do so to share information about astrophysics in general, rather than specifically to discuss or promote their own research. It is, however, noteworthy to highlight the fact that astrophysicists, no matter their publishing behavior or tweeting behavior, have plenty of conversational connections with Twitter users of varying roles and professions.

A small world graph emerged from the visualization of the conversational connections (outgoing ego network based on the conversations initiated or usernames mentioned by the astrophysicists) between the researched astrophysicists and other Twitter users as indicated by dense local clusters and short distances between the nodes in the graph. A closer look at the conversational connections revealed some differences in the connections in the clusters. One cluster clearly had more conversational connections to other astrophysicists (outside the group of astrophysicists studied in this research), while another cluster had more connections to science communicators. One of the clusters had more connections to researchers in other research areas than astrophysics or astronomy, possibly indicating an interdisciplinary component to their research. Another cluster had more connections to students and teachers, possibly suggesting that these astrophysicists use Twitter more for educational purposes. The results showed a great variation in the professional composition of the clusters created by conversational connections. Interestingly the community detection did not just discover clusters of people with more frequent conversational connections to each other, but it also discovered clusters of people with the same professional roles. It is not clear what roles these connections play, if any, between those using Twitter for personal reasons as compared to those using Twitter for professional reasons; more research is needed to examine any differences.

Although the conversational connections revealed distinct clusters, it is striking that these clusters often use the same words and hashtags when tweeting. We have shown that the professional roles in the clusters are very different, yet the content of their tweets are very similar. The differences in the professional composition of the clusters suggest that although the content of the tweets are very similar, the motivations for tweeting are different because the intended target audiences are different. For altmetric research this raises some questions: As a measure of research visibility, are all tweets equal? Are all mentions equal? Are the altmetric indicators always created in a specific type of conversational context? What affordances and norms do scholars utilize to distinguish personal and professional tweets and can altmetric indicators discriminate between the two roles?

Another question that arises from the results is why the clusters are not more connected to each other if they are interested in the same topics? One reason for that might be that mentioning someone in a tweet reflects a real-world network and are simply conversations between friends or colleagues rather than pure conversational networks based on topics, like Gruzd, Wellman, and Takhteye [48] have suggested. The content of the tweets also suggests another reason. The majority of the tweets do not contain jargon specific to astrophysics, but rather astrophysics on a more general level. This suggests that the astrophysicists have framed their communications so that they can connect with diverse audiences, as suggested for instance by Niset [7] and Groth and Gurney [6]. More specialized discussions between astrophysicists and astronomers might appear in the Astronomers group on Facebook mentioned above. The affordances of Twitter, including the limitation of 140 characters, the use of hashtags, mentions, and retweets, and the limited profile information, may also contribute to the disconnect between clusters because of the way in which the tweets are framed by the various actors in the network; more research is needed on Twitter affordance use and framing.

The sentiment analysis of tweets resulted in low sentiment scores for positive sentiments and negative sentiments for all clusters, although there was one cluster (Mod3) that discussed budget cuts in science funding and was perhaps expected to produce negative sentiments. A study of brand related tweets [49] found that two thirds of tweets contain positive sentiments, suggesting that Twitter users rather tend to tweet positive expressions than negative. That phenomenon might be supported by our next finding, that a connection was discovered between the numbers of tweets sent in a cluster and the sentiment of those tweets. The results showed that in the clusters where more tweets were sent the tweets tend to be more neutral, in contrast to somewhat more positive tweets in the clusters where fewer tweets were sent. Since [26] could show that the expression of sentiments does not depend on the tweeting frequency, this suggests that those astrophysicists that tweet frequently do so mainly to share information, not to express their own opinion. However, given that the analyzed clusters varied in size and tweeting frequency we present tendencies instead of statistically significant correlations.

The present study is not completely without limitations. The research is limited by its small sample size (tweets from 32 astrophysicists) and, as such, gives some first insights into the astrophysicists tweeting behavior for scholarly communication. In future work we will extend the analysis to researchers from different disciplines to examine whether there are discipline-specific conversation strategies in scholarly communication on Twitter. In content analysis and manual coding of objects, the coding should ideally be done by at least two people and inter-coder reliability (e.g. Cohen’s kappa) should be calculated. Coding of the usernames by professional types in this research was done by one author based on information found on the Twitter profiles of each user. Although in most cases the coding was fairly straightforward with not much room for interpretation (e.g. “Astrophysics Professor at X”), there were some ambiguous cases (e.g. “Assistant professor in astrophysics, science blogger, teacher”). In cases where more than one role could have fitted the user, we chose to code the user based on the first role the user mentioned. Another limitation that needs to be acknowledged is the use of the community detection. We used the built-in community detection in Gephi [43], [44], but there are other algorithms that could have been used too and that could have taken into account that a user may simultaneously belong to two or more different communities or clusters. Although this was beyond the scope of this research, an interesting future direction could be to focus on the users that have multiple roles and that simultaneously belong to two or more clusters.
